# Molecular Signaling in Stroke

**DOI:** 10.3390/ijms24065975

**Published:** 2023-03-22

**Authors:** Naseem Akhter, Saif Ahmad

**Affiliations:** 1 Department of Neurology, Henry Ford Health System, Detroit, MI 48202, USA; nakhter2@hfhs.org; 2 Department of Neurosurgery and Translational Neuroscience, Barrow Neurological Institute, SJHMC, Dignity Health, Phoenix, AZ 85013, USA

We have reached the end of the Special Issue on Molecular Signaling in Stroke in *IJMS*. This issue has brought together a diverse set of research articles that highlight the importance of understanding the molecular signaling pathways involved in stroke. The studies presented have shed light on the molecular mechanisms that drive stroke and have provided insights into potential therapeutic targets for this disease.

The use of rodent models in stroke research has significantly advanced our understanding of stroke pathophysiology and has contributed to the development of new therapies and treatment strategies. However, metabolic and transcriptomic variability in different brain regions must be considered [1,2]. Filippenkov et al., 2022 [3], investigates the genetic response in the rat brain after a focal stroke using both contralateral and sham-operated controls. They found that genes involved in inflammation and immune response were upregulated in both the contralateral and ipsilateral hemispheres, suggesting a bilateral genetic response to stroke. They also found that genes related to axonal growth and synaptic plasticity were upregulated in the contralateral hemisphere, but not in the ipsilateral hemisphere or sham-operated controls, indicating a specific response to stroke in the contralateral hemisphere. The findings suggest that both contralateral and sham-operated controls should be used in stroke studies to better understand the bilateral genetic response to stroke.

After an ischemic stroke, the brain undergoes a complex cascade of biochemical and molecular changes, including alterations in calcium (Ca^2+^) signaling [4]. Ca^2+^ plays a crucial role in various cellular processes, including neurotransmitter release, gene expression, excitotoxicity, oxidative stress, inflammation and cell death. Kourti et al., 2022 [5], explored the role of calcium signaling in the activation of Akt, a protein kinase involved in cell survival, in glucose-deprived human neuroblastoma cells (SH-SY5Y). The authors found that glucose deprivation leads to an increase in intracellular calcium concentration, which in turn activates Akt through a mechanism involving the calcium-dependent phosphatase calcineurin. The authors also found that blocking calcium entry into the cells through the L-type calcium channel or the store-operated calcium channel inhibits Akt activation and promotes cell death. Overall, the study suggests that calcium signaling is an important regulator of cell survival in glucose-deprived conditions and may represent a potential target for therapeutic intervention in neurodegenerative diseases associated with glucose metabolism dysfunction.

Early studies have focused on the role of non-coding RNAs by showing that targeting specific microRNAs could potentially be a new avenue for developing effective stroke therapies [6,7]. Voelz et al., 2022 [8], studied the changes in miRNA expression in various tissues after an episode of transient focal cerebral ischemia (TFCI). The authors analyzed the miRNA expression in different brain regions, blood serum, liver, and spleen of rats that underwent TFCI and compared it to a control group. They found that TFCI caused significant alterations in the miRNA expression in all the tissues studied, with some miRNAs showing tissue-specific expression patterns. In addition, the study identified novel miRNA alterations in the blood serum, liver, and spleen that may be related to the systemic response to cerebral ischemia. The study suggests that TFCI can lead to systemic changes in miRNA expression that may have implications for post-stroke recovery and also could potentially serve as biomarkers for stroke.

Another key theme in this issue was the importance of early diagnosis and treatment in stroke management. Several studies have emphasized the need for the rapid and accurate diagnosis of stroke to ensure the timely administration of appropriate treatments, such as thrombolytic therapy [9,10]. Sikora et al., 2022 [11], provide a comprehensive review of the importance of platelet response during antiplatelet treatment after an ischemic stroke by highlighting the critical role of platelets in thrombus formation and emphasize the need for effective antiplatelet therapy to prevent recurrent ischemic events. The article reviews the current evidence regarding the efficacy and safety of antiplatelet therapy in patients with ischemic stroke. The authors discuss the different classes of antiplatelet drugs, including aspirin, P2Y12 inhibitors, and glycoprotein IIb/IIIa inhibitors, and highlight the importance of an individualized treatment based on the patient’s risk factors and response to therapy. The authors also discuss the various methods used to assess the platelet response to antiplatelet therapy, including platelet function tests and genetic testing further emphasizing the need for standardized protocols for platelet function testing and the importance of interpreting the results in the context of the patient’s clinical history.

Overall, the article provides a comprehensive overview of the importance of platelet response during antiplatelet treatment after ischemic stroke and highlights the need for individualized treatment based on the patient’s risk factors and response to therapy.

Intracerebral hemorrhage (ICH) can impact patient prognosis and survival through two significant factors: inflammation and apoptosis. Toll-like receptor 4 (TLR4) plays a pivotal role in activating the inflammatory pathway, which results in the production and release of inflammatory factors leading to neuronal death [12,13]. Lua et al., 2022 [14] discusses the potential role of soluble Toll-like receptors 2 and 4 (sTLR2 and sTLR4) as therapeutic agents for stroke and brain hemorrhage. The authors explain that sTLR2 and sTLR4 can bind to pathogen-associated molecular patterns (PAMPs) and damage-associated molecular patterns (DAMPs) to neutralize their effects on the immune system. The authors describe the pathophysiology of stroke and brain hemorrhage, highlighting the role of inflammation in these conditions, and argue that sTLR2 and sTLR4 could reduce inflammation and improve outcomes in stroke and brain hemorrhage. The authors review preclinical studies that have investigated the use of sTLR2 and sTLR4 in animal models of stroke and brain hemorrhage and report that sTLR2 and sTLR4 have been shown to reduce inflammation, decrease brain damage, and improve functional outcomes in these models. The authors also discuss the potential clinical applications of sTLR2 and sTLR4, noting that while clinical studies have not yet been conducted, sTLR2 and sTLR4 are promising therapeutic agents for stroke and brain hemorrhage due to their ability to modulate the immune response and reduce inflammation. Overall, the article suggests that sTLR2 and sTLR4 have the potential to be effective therapeutic agents for stroke and brain hemorrhage, and calls for further research to investigate their clinical applications.

Overall, the research presented in this Special Issue underscores the complex nature of strokes and the importance of a multidisciplinary approach to stroke research. By understanding the molecular signaling pathways involved in strokes, we can develop new and innovative approaches for preventing and treating this devastating disease. We would like to extend our sincere thanks to all the authors, reviewers, and editors who contributed to this Special Issue. We hope that the articles presented here will inspire further research and innovation in the field of strokes and molecular signaling.
